# Comparison of clinical outcomes between the depot gonadotrophin-releasing hormone agonist protocol and gonadotrophin-releasing hormone antagonist protocol in normal ovarian responders

**DOI:** 10.1186/s12884-021-03849-8

**Published:** 2021-05-11

**Authors:** Min Xia, Jie Zheng

**Affiliations:** Reproductive Center, Women and Children’s Hospital of Hubei Province, No. 745, Wuluo Road, Hongshan District, 430070 Wuhan, China

**Keywords:** Gonadotrophin-releasing hormone antagonist, Gonadotrophin-releasing hormone agonist, Frozen-thawed embryo, Clinical pregnancy rate

## Abstract

**Background:**

The gonadotrophin-releasing hormone (GnRH) antagonist protocol has some advantages, such as a simple method, short medication duration, and low incidence of ovarian hyperstimulation syndrome, but whether the GnRH antagonist protocol is suitable for normal ovarian responders has been controversial. We compared the clinical outcomes of fresh and frozen-thawed transfer cycles between the depot GnRH agonist protocol and GnRH antagonist protocol in normal ovarian responders.

**Methods:**

Data of normal ovarian responders who underwent in vitro fertilization-embryo transfer (IVF-ET) or intracytoplasmic sperm injection-embryo transfer (ICSI-ET) between January 2017 and December 2018 in our hospital were retrospectively analysed. In this study, there were 1119 fresh transfer cycles, including 502 GnRH antagonist cycles (GnRH antagonist group) and 617 depot GnRH agonist cycles (depot GnRH agonist group), as well as 468 frozen-thawed transfer cycles, includng 191 GnRH antagonist cycles (GnRH antagonist group) and 277 depot GnRH agonist cycles (depot GnRH agonist group). The clinical outcomes were compared between the GnRH antagonist group and the depot GnRH agonist group.

**Results:**

With the fresh transfer cycles, there were no statistically significant differences in the anti-Mullerian hormone level, number of transferred embryos or high-quality embryo rate between the two groups. The total dosage of gonadotropin (Gn), duration of Gn stimulation, number of oocytes retrieved, clinical pregnancy rate and incidences of moderate and severe ovarian hyperstimulation syndrome (OHSS) were significantly lower but the abortion rate was significantly higher in the GnRH antagonist group than in the depot GnRH agonist group (all *P* < 0.05). With the frozen-thawed transfer cycles, there were no statistically significant differences in the number of transferred embryos, clinical pregnancy rate or abortion rate between the two groups (all *P* > 0.05).

**Conclusions:**

With the fresh transfer cycles, the GnRH antagonist protocol had a lower clinical pregnancy rate and lower incidences of moderate and severe OHSS than the depot GnRH agonist protocol, but with the frozen-thawed transfer cycles, both protocols had similar clinical pregnancy rates. These results remain to be further confirmed through large-sample, prospective, randomized and controlled studies.

## Background

With the rapid development of assisted reproductive technology (ART), it is important for every reproductive doctor to make safe and efficient ovulation induction protocols for patients. Theoretically, the gonadotrophin-releasing hormone (GnRH) antagonist protocol has some advantages, such as a simple method, short medication duration, and low incidence of ovarian hyperstimulation syndrome (OHSS). However, at present, the GnRH antagonist protocol is mainly used in high or poor ovarian responders. Whether the GnRH antagonist protocol is suitable for normal ovarian responders has been controversial [1]. A meta-analysis including five randomized controlled trials on normal ovarian responders showed that the live birth rate was significantly lower with the GnRH antagonist protocol than with the depot GnRH agonist protocol, suggesting that the depot GnRH agonist protocol is relatively suitable for normal ovarian responders [2]. A retrospective study showed no significant difference between the GnRH antagonist protocol and the depot GnRH agonist protocol in clinical and ongoing pregnancy rates, but the antagonist regimen significantly relieved patient discomfort and reduced the economic pressure on patients [3]. Therefore, the aim of this study was to provide more clinical references for the selection of ovulation induction protocols in normal ovarian responders through a retrospective comparison of clinical outcomes between the GnRH antagonist protocol and depot GnRH agonist protocol.

## Methods

This research was performed in accordance with the Declaration of Helsinki, and relevant guidelines and regulations. All study methods were also approved by the Institutional Review Board and Ethics Committee of the Women and Children′ Hospital of Hubei Province [2016]IEC[BL003]. The patients described in this study gave written informed consent to participate.

### Study population

Normal ovarian responders who underwent fertilization-embryo transfer (IVF-ET) or intracytoplasmic sperm injection-embryo transfer (ICSI-ET) in our hospital between January 2017 and December 2018, were retrospectively studied. In this study, there were 1119 fresh transfer cycles, including 502 GnRH antagonist cycles (GnRH antagonist group) and 617 depot GnRH agonist cycles (depot GnRH agonist group), as well as 468 frozen-thawed transfer cycles, includng 191 GnRH antagonist cycles (GnRH antagonist group) and 277 depot GnRH agonist cycles (depot GnRH agonist group). The inclusion criteria were as follows: (1) age < 35 years; (2) basal follicle-stimulating hormone (FSH) level < 10 IU/L; (3) anti-Mullerian hormone (AMH) level > 1.2 µg/L; (4) basal antral follicle count (AFC) ≥ 5 and (5) IVF-ET or ICSI-ET. The exclusion criteria were as follows: (1) patients with polycystic ovary syndrome (PCOS); (2) patients with ovarian insufficiency; (3) patients with an abnormal uterine cavity that affected embryo implantation; and (4) patients who required a genetic diagnosis before embryo implantation.

### Ovulation induction protocols

Depot GnRH agonist protocol: On the first to third days of the menstrual cycle, antral follicles were observed by vaginal B-mode ultrasound. If the diameters of all antral follicles were less than 10 mm, 3.75 mg of diphereline (GnRHa, Ipsen Pharmaceutical Co., Ltd.) was injected intramuscularly. Twenty-eight days later, if the laboratory examination indicated a luteinizing hormone (LH) level < 5 mIU/ml and an oestradiol (E_2_) <50 pg/ml, as well as B-mode ultrasound showed an endometrial thickness < 5 mm and a follicular diameter < 10 mm, 150 ~ 300 IU of gonadotrophin (Gn) was first given, and then the dose of Gn was adjusted according to follicular development and serum endocrine. When the follicular diameter reached 18 mm in 2 or more ovarian follicles, or reached 17 mm in 3 or more ovarian follicles; 250 µg of ovidrel was injected subcutaneously. Thirty-four to thirty-six hours later, oocyte retrieval was performed. If there was no abnormal condition, one or two fresh embryos were transferred 3 days after oocyte retrieval. Other embryos were frozen directly or after blastocyst-culture.

GnRH antagonist protocol: On the first to third days of the menstrual cycle, 150 ~ 300 IU of Gn was first given, and then the dose of Gn was adjusted and an antagonists (0.25 mg of cetrorelix, Merck, Switzerland; or 0.25 mg of ganirelix, MRK, Netherlands) was given according to follicular development until the day of human chorionic gonadotropin (hCG) administration. When the follicular diameter reached 18 mm in 2 or more ovarian follicles, or reached 17 mm in 3 or more ovarian follicles; 250 µg of ovidrel or 0.2 mg of diphereline (Ipsen Pharmaceutical Co., Ltd.) combined with 2000 IU of hCG was injected subcutaneously. Thirty-four to thirt-six hours later, oocyte retrieval was performed. If there was no abnormal condition, one or two fresh embryos were transferred 3 days after oocyte retrieval. Other embryos were frozen directly or after blastocyst-culture.

### Endometrial preparation for frozen-thawed transfer cycles

Natural cycle: Natural cycle was suitable for patients with regular menstrual cycles. When transvaginal B-mode ultrasound indicated that the dominant follicle diameter was 18 mm, 6000–10,000 IU of hCG was injected. Four days later, one or two embryos were transferred; alternatively, 6 days later, one or two blastocysts were transferred.

Artificial cycle: Artificial cycle was suitable for patients with irregular menstrual cycles. From the 3rd day of menstruation, patients took progynova (2–6 mg/d, Bayer Company, Germany) until the endometrial thickness was ≥ 8 mm, progesterone (40–60 mg/d, Guangzhou Baiyunshan Pharmaceutical Industry, Guangzhou, China) was injected intramuscularly. Three days later, one or two embryos were transferred; alternatively, 5 days later, one or two blastocysts were transferred.

### Outcome measures

Clinical outcomes included the clinical pregnancy rate, abortion rate and incidences of moderate and severe OHSS. Clinical pregnancy was diagnosed by the ultrasonographic visualization of one or more gestational sacs or a fetal heartbeat 4–6 weeks after embryo transfer. Moderate and severe OHSS were diagnosed according to guidelines on prevention and treatment of moderate and severe OHSS [4]. Clinical pregnancy rate = number of clinical pregnancy cycles/number of embryo transfer cycles; abortion rate = number of abortion cycles/number of clinical pregnancy cycles; the incidences of moderate and severe OHSS = number of moderate and severe OHSS cycles/total number of oocyte retrieval cycles.

### Grading system used to evaluate embryo quality

According to Peter’s standards [5], day 3 embryos were divided into the following 4 grades: Grade I, the blastomeres had even sizes, regular shapes and intact zona pellucidae, and cell debris was less than 10 %; Grade II, the blastomeres had a slightly irregular morphology, the cytoplasm might have granulation, and cell debris was between 10 and 20 %; Grade III, the blastomeres were uneven in size and irregular in shape, the cytoplasm had obvious granulation, and cell debris was between 20 and 50 %; and Grade IV, the blastomeres were severely uneven in size and severely irregular in shape, the cytoplasm had a lot of granulation, and cell debris was more than 50 %. The high-quality embryos included grade I and grade II embryos.

### Statistical analysis

A power analysis was performed to determine the number of patients necessary to distinguish significant differences between two groups, and the sample size to detect a difference was 60 patients per group (α = 0.05 and 1-β = 0.9). Statistical analysis was performed using SPSS19.0 software (IBM, Armonk, New York, USA). The measurement data are expressed as the mean ± standard deviation (x ± s), and were analysed using an independent samples *t* test. Counting data are expressed as the rate (%) and were analysed using the χ^2^ test or Fisher’s exact test. Statistical significance was established at *P* < 0.05.

## Results

### General data

In this study, there were 1119 fresh transfer cycles, including 502 GnRH antagonist cycles and 617 depot GnRH agonist cycles. There were no statistically significant differences in the mean age, infertility duration, infertility causes, body mass index (BMI), AMH or infertility-related factor constitute between the two groups (all *P* > 0.05, Table 1).

**Table 1:** Comparison of general data between the two groups

Groups	GnRH antagonist group	Depot GnRH agonist group	*P* values
Cycles (n)	502	617	
Maternal age (year)	28.75 ± 5.44	29.59 ± 4.38	>0.05
Infertility duration	3.79 ± 3.4	3.25 ± 2.6	>0.05
Primary infertility rate	48.23 %	51.42 %	
Infertile causes (%)			
Male factors	19.52 %	20.97 %	>0.05
Tubal factors	61.16 %	65.15 %	>0.05
Couples′ factors	19.32 %	13.88 %	>0.05
BMI	22.45 ± 3.37	23.98 ± 5.12	>0.05
AMH	3.39 ± 1.41	3.68 ± 1.38	>0.05

Notes: *GnRH* gonadotrophin-releasing hormone, *GnRHa* gonadotrophin-releasing hormone agonist, *BMI* body mass index, *AMH* anti-mullerian hormone

### Clinical outcomes of fresh transfer cycles

There were no statistically significant differences in the number of transferred embryos or high-quality embryo rate between the two groups (all *P* > 0.05). However, the total dosage of Gn, duration of Gn stimulation, number of oocytes retrieved, clinical pregnancy rate and incidences of moderate and severe OHSS were significantly lower but the abortion rate was significantly higher in the GnRH antagonist group than in the depot GnRH agonist group (all *P* < 0.05, Table 2).

**Table 2:** Comparison of clinical outcomes between the two groups in fresh cycles

Groups	GnRH antagonist group	Depot GnRH agonist group	*P* values
Cycles (n)	502	617	
Duration of Gn stimulation (day)	10.23 ± 2.39	12.94 ± 1.94	<0.05
Total dosage of Gn (IU)	2423.16 ± 935.68	3046.44 ± 876.29	<0.05
Number of oocytes retrieved	9.97 ± 4.1	16.23 ± 3.8	<0.05
Number of transferred embryos	1.85 ± 0.78	1.87 ± 0.83	>0.05
High-quality embryo rate (%)	61.47 %(2616/4256)	56.24 %(4872/8663)	>0.05
Clinical pregnancy rate (%)	37.27 %(131/352)	54.89 %(170/310)	<0.05
Abortion rate (%)	18.32 %(24/131)	10.58 %(18/170)	<0.05
Moderate and severe OHSS (%)	8.37 %(42/502)	13.12 %(81/617)	<0.05

Notes: *GnRH* gonadotrophin-releasing hormone, *GnRHa* gonadotrophin-releasing hormone agonist, *Gn* gonadotropin, *OHSS* ovarian hyperstimulation syndrome

### Clinical outcomes of frozen-thawed transfer cycles

There were no statistically significant differences in the number of transferred embryos, clinical pregnancy rate or abortion rate between the two groups (all *P* > 0.05, Tables 3 and 4).

**Table 3:** Comparison of clinical outcomes between the two groups in frozen-thawed Day 3 embryo transfer

Groups	GnRH antagonist group	Depot GnRH agonist group	*P* values
Cycles (n)	78	79	
Number of transferred embryos	2.0 ± 0.4	1.9 ± 0.5	>0.05
Clinical pregnancy rate (%)	47.43 %(37/78)	51.89 %(41/79)	>0.05
Abortion rate (%)	13.51 %(5/37)	14.63 %(6/41)	>0.05

Notes: *GnRH* gonadotrophin-releasing hormone, *GnRHa* gonadotrophin-releasing hormone agonist

**Table 4:** Comparison of clinical outcomes between the two groups in frozen-thawed blastocyst transfer

Groups	GnRH antagonist group	Depot GnRH agonist group	*P* values
Cycles (n)	113	198	
Number of transferred embryos	1.81 ± 0.62	1.74 ± 0.78	>0.05
Clinical pregnancy rate (%)	60.18 %(68/113)	68.68 %(135/198)	>0.05
Abortion rate (%)	11.76 %(8/68)	12.59 %(17/135)	>0.05

Notes: *GnRH* gonadotrophin-releasing hormone, *GnRHa* gonadotrophin-releasing hormone agonist

### Cumulative pregnancy rates in the two groups

The cumulative pregnancy rate was 74.30 % in the GnRH antagonist group and 79.09 % in the depot GnRH agonist group. Although the cumulative pregnancy rate was slightly lower in the GnRH antagonist group than in the depot GnRH agonist group, the dfference was no statistically significant (*P* > 0.05, Table 5).

**Table 5:** Comparison of cumulative pregnancy rate between the two groups

Groups	GnRH antagonist group	Depot GnRH agonist group	*P* values
Number of cycles	502	617	
Number of frozen-thawed cycles	344 (68.53 %,344/502)	463 (75.04 %,463/617)	>0.05
Cumulative pregnancy rate	74.30 % (373/502)	79.09 % (488/617)	>0.05

## Discussion

There is no consensus on which ovulation induction protocol is the best for normal ovarian responders, so we compared the clinical outcomes of fresh and frozen-thawed transfer cycles between the depot GnRH agonist protocol and GnRH antagonist protocol in normal ovarian responders to provide more references for clinical practice.

The GnRH antagonist protocol has been widely used in high or poor ovarian responders because it is characterized by a short duration of ovarian stimulation and a low Gn dosage [6]. With improvements in GnRH antagonist protocol, increasing attention has been given to its application in normal ovarian responders. In clinical practice, patient age, baseline FSH, AFC and AMH are usually used to predict the ovarian response to ovulation induction. Except for patients with a high ovarian response or diminished ovarian reserve, patients who are less than 35 years old and have a basal FSH levle < 10 IU/L, AFC ≥ 5 and AFC level ≥ 1.2 µg/L are regarded as normal ovarian responders [7–9]. Therefore, in this study, we selected normal ovarian responders based on the above items, and retrospectively analysed the clinical outcomes of the two different protocols: the depot GnRH agonist protocol and GnRH antagonist protocol.

Our results showed that the number of oocytes retrieved and clinical pregnancy rate were significantly higher but the abortion rate was significantly lower in the depot GnRH agonist group than in the GnRH antagonist group with fresh transfer cycles. The main reasons for the higher pregnancy rate of the depot GnRH agonist protocol with fresh transfer cycles are as follows: ① According to the theory of the “LH therapeutic window “, a high concentration of LH can lead to follicular atresia. The depot GnRH agonist protocol may increase the number of oocytes retrieved because it can decrease LH level and reduce follicular atresia. Gao et al. [10] reported that an increased dosage and duration of GnRHa could significantly increase the number of retrieved oocytes and the rate of MII oocytes. ② Improving the rate of high-quality embryos: The depot GnRH agonist protocol can decrease interleukin-1 (IL-1), interleukin-8 (IL-8), vascular endothelial growth factor, tumour necrosis factor, oestrogen receptor and aromatase P450, improving oocyte and embryo quality [11, 12]. However, in this study, there was no significant difference in the high-quality embryo rate between the depot GnRH agonist protocol and the GnRH antagonist group. ③ The depot GnRH agonist protocol may be conducive to improving endometrial receptivity. It has been reported that homeobox gene (HOX) A10, pinopodes and integrin are used as markers to evaluate endometrial receptivity, and these markers are positively associated with endometrial receptivity [13]. GnRHa may increase the number of pinopodes and the level of integrin, improving endometrial receptivity and the pregnancy rate. Moreover, an increased dosage and duration of GnRHa can inhibit irregular endometrial growth, local inflammatory reactions and autoantibody production and decrease the levels of tumor necrosis factor and interleukin-1 in body fluid [14], which are conducive to embryo implantation.

Ding et al. [15] reported that the depot GnRH agonist protocol could improve endometrial receptivity and the clinical pregnancy rate, but it also increased the OHSS risk, consistent with our results.

With oocyte donation cycles, there was no significant difference in embryo development, the embryo implantation rate or the pregnancy rate between the depot GnRH agonist group and the GnRH antagonist group [16]. Our results also indicated no significant differences in the high-quality embryo rate, pregnancy rate or abortion rate with frozen-thawed transfer cycles between the two groups, but with fresh transfer cycles, the pregnancy rate was significantly lower in the GnRH antagonist group than in the depot GnRH agonist group. The above results indirectly suggest that the GnRH antagonist protocol may affect endometrial receptivity, which is consistent with the results reported by Jin and Xu [17].

The GnRH antagonist protocol is usually used in high or poor ovarian responders. However, our results suggest that the GnRH antagonist protocol is also suitable for normal ovarian responders. Our results demonstrated that with fresh transfer cycles, the pregnancy rate was significantly lower in the GnRH antagonist group than in the depot GnRH agonist group, but close attention to the LH level on hCG day might allow the pregnancy rate of fresh transfer cycles to reach a similar level between the GnRH antagonist and depot GnRH agonist protocols [18]. We should try to improve endometrial receptivity to increase the pregnancy rate of fresh embryo transfer with the GnRH antagonist protocol in normal ovarian responders because the GnRH antagonist protocol has a lower OHSS rate than the depot GnRH agonist protocol and similar high-quality embryo rate.

Our results indicated no significant differences in the high-quality embryo rate, pregnancy rate and abortion rate with frozen-thawed transfer cycles between the two groups. The incidences of moderate and severe OHSS were significantly lower in the GnRH antagonist group than in the depot GnRH agonist group. Therefore, to ensure the pregnancy rate and decrease the incidences of moderate and severe OHSS, it is better for normal ovarian responders to undergo frozen-thawed embryo transfer after GnRH antagonist protocol-induced ovulation.

The limitation of this study was that it was a retrospective study. The imbalanced comparisons may also have weakened the quality of the evidence presented herein.

## Conclusions

With the fresh transfer cycles, the GnRH antagonist protocol had a lower clinical pregnancy rate and lower incidences of moderate and severe OHSS than the depot GnRH agonist protocol, but with the frozen-thawed transfer cycles, both protocols had similar clinical pregnancy rates. These results remain to be further confirmed through large-sample, prospective, randomized and controlled studies.

## Data Availability

The datasets used and/or analysed during the current study are available from the corresponding author on reasonable request.

## Abbreviations

IVF-ET: In vitro fertilization-embryo transfer
ICSI-ET: Intracytoplasmic sperm injection-embryo transfer
GnRHa: Gonadotrophin-releasing hormone agonist
OHSS: Ovarian hyperstimulation syndrome
ART: Assisted reproductive technology
BMI: Body mass index
IL-1: Interleukin-1
IL-8: Interleukin-8
